# Supplementary material for: Three phylogenetic groups have driven the recent population expansion of Cryptococcus neoformans
Source: Nat Commun. 2019 May 2;10:2035. doi: 10.1038/s41467-019-10092-5 (PMC6497710; doi:10.1038/s41467-019-10092-5)
Supplement: Supplementary file 1 — Supplementary Information [file 41467_2019_10092_MOESM1_ESM.pdf]

Supplementary Information for 'Three phylogenetic groups have driven the recent population expansion of *Cryptococcus neoformans*', Ashton et al.

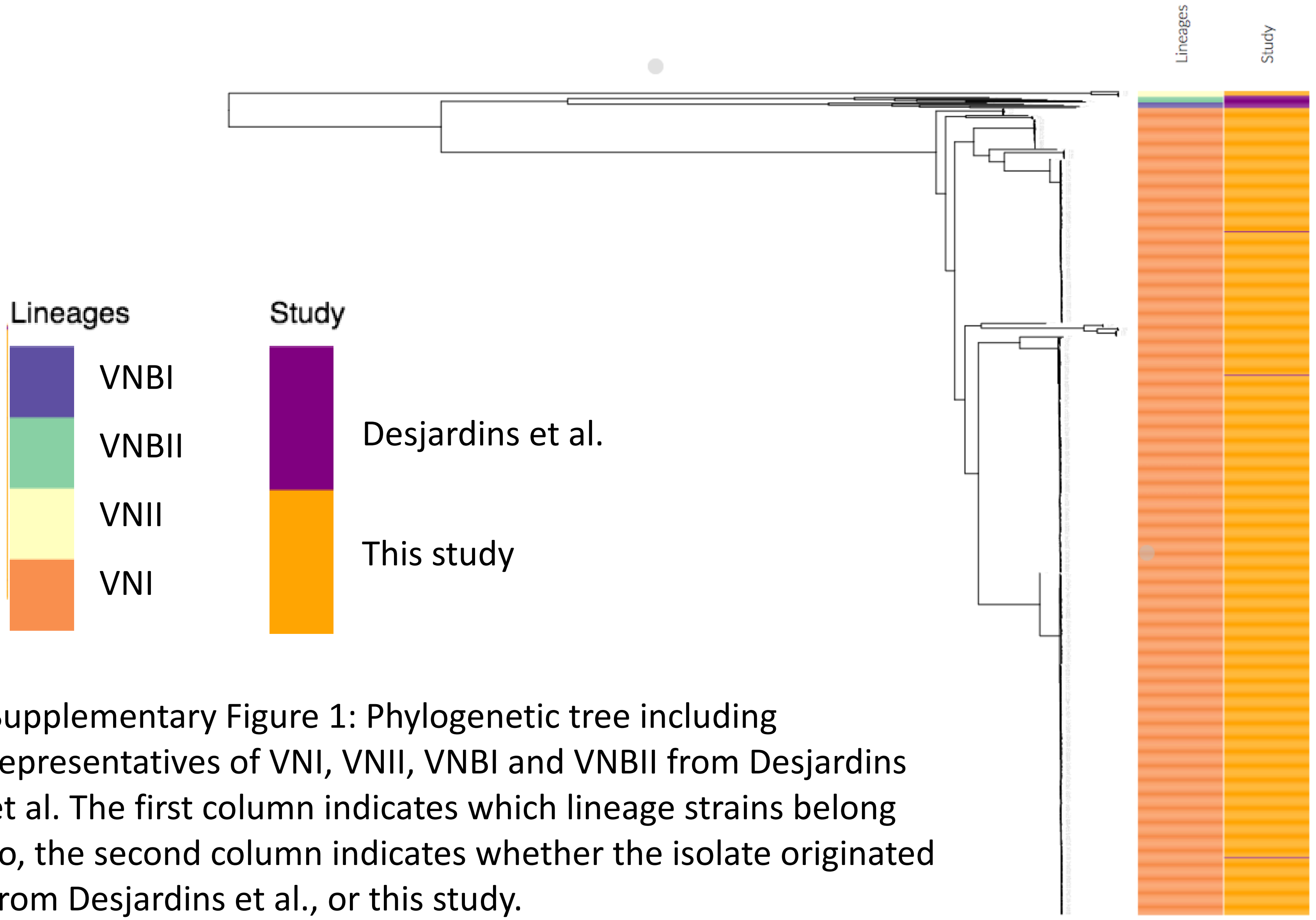

Supplementary Figure 1: Phylogenetic tree including representatives of VNI, VNII, VNBI and VNBII from Desjardins et al. The first column indicates which lineage strains belong to, the second column indicates whether the isolate originated from Desjardins et al., or this study.

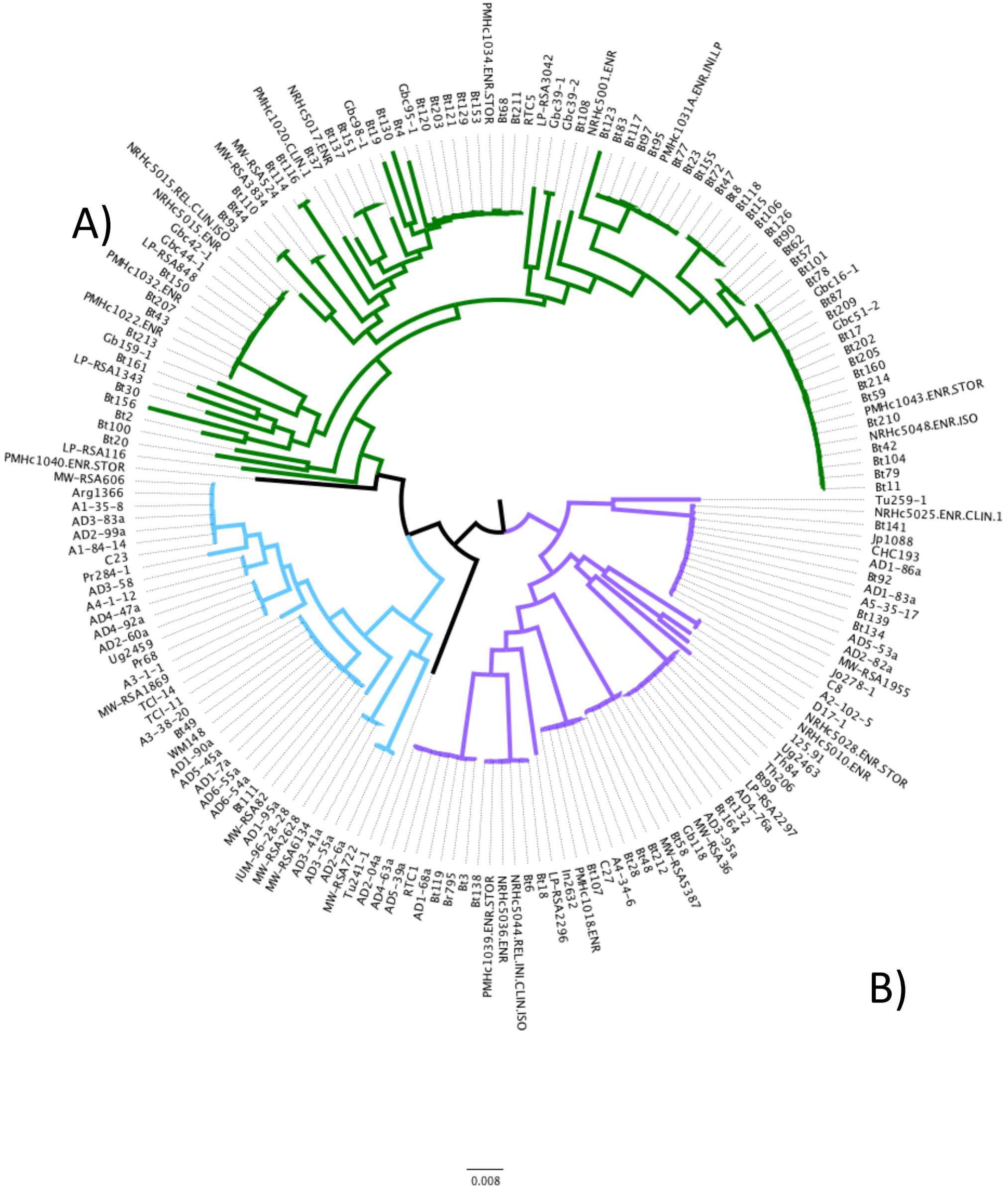

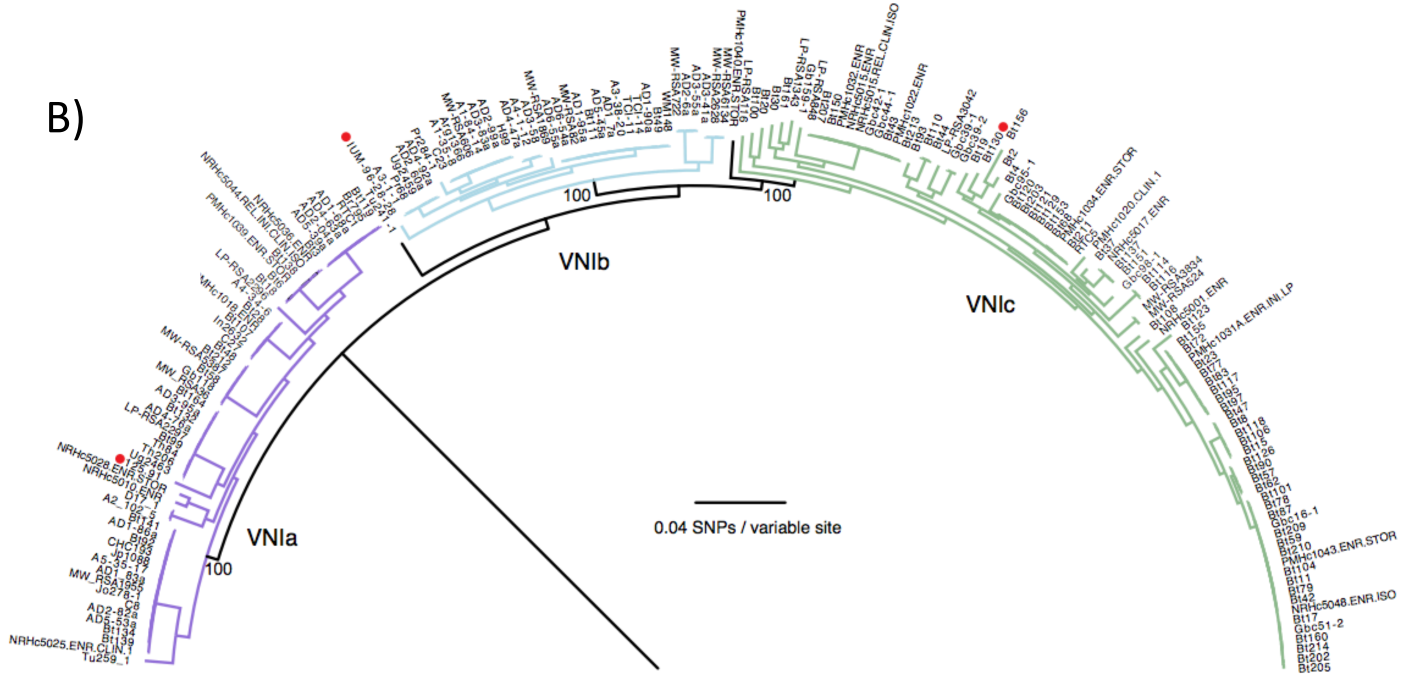

Supplementary Figure 2: A) our phylogenetic analysis of Desjardins et al. data from VNI strains B) Supplementary Figure S1 from Desjardins et al. 2017.

A)

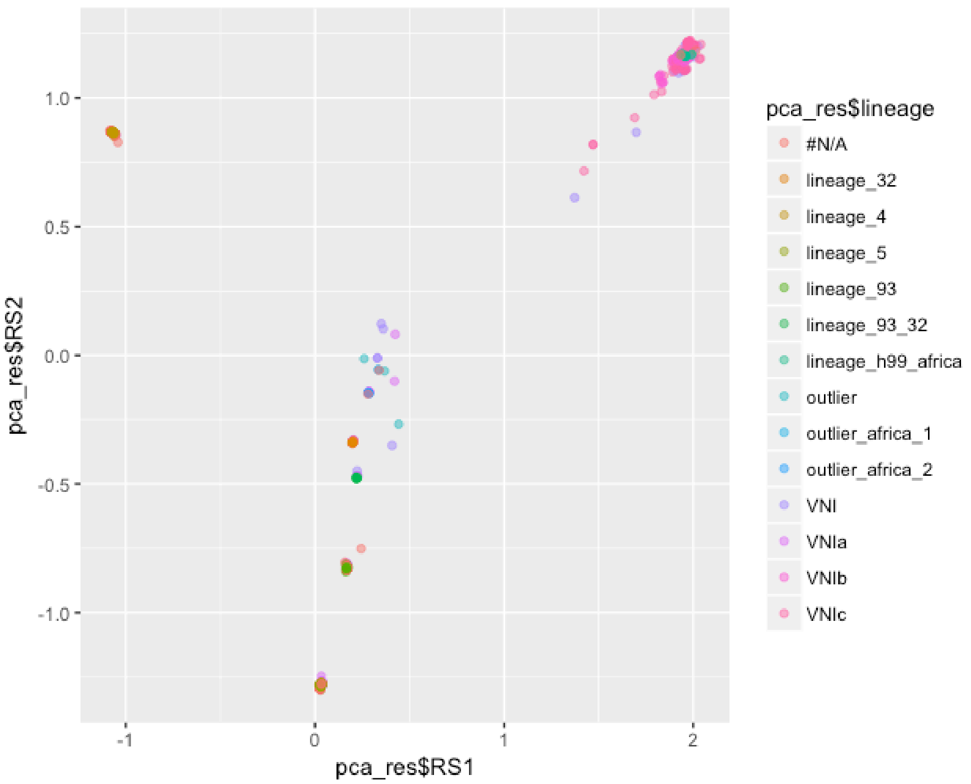
pca_res$RS2
pca_res$RS1
1.0
0.5
0.0
-0.5
-1.0
-1
0
1
2
pca_res$lineage
#N/A
lineage_32
lineage_4
lineage_5
lineage_93
lineage_93_32
lineage_h99_africa
outlier
outlier_africa_1
outlier_africa_2
VNI
VNIa
VNIb
VNIc

B)

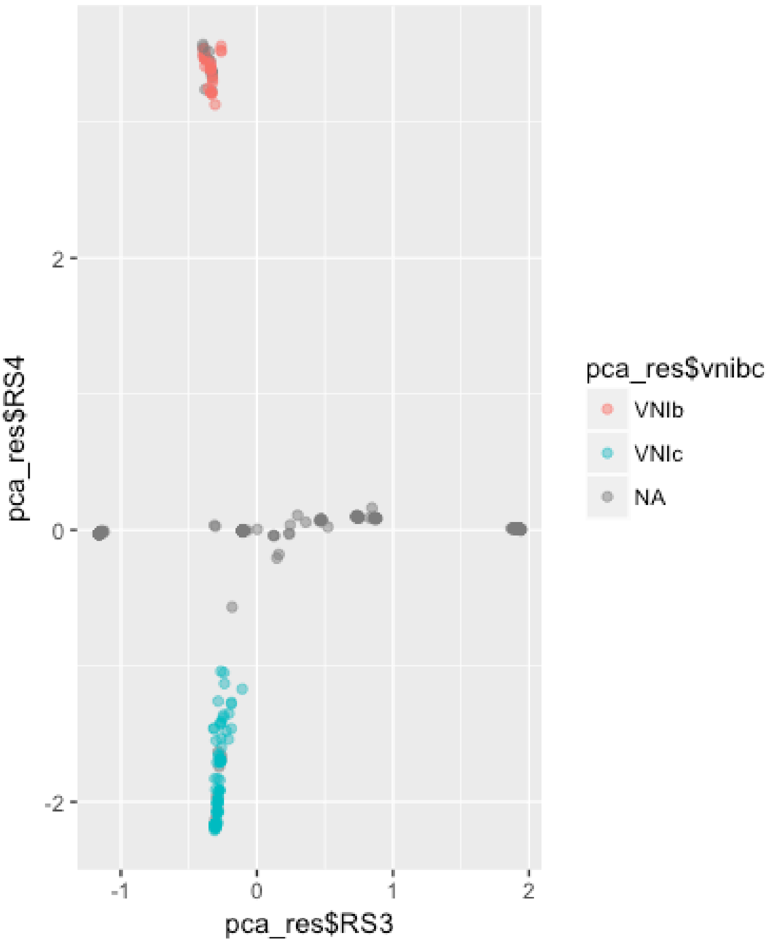
pca_res$RS4
pca_res$RS3
2
0
-2
-1
0
1
2
pca_res$vnibc
VNIb
VNIc
NA

Supplementary Figure 3: (A) clustering of the first and second PCs, colour corresponds to k-means cluster (B) clustering of the third and fourth PCs

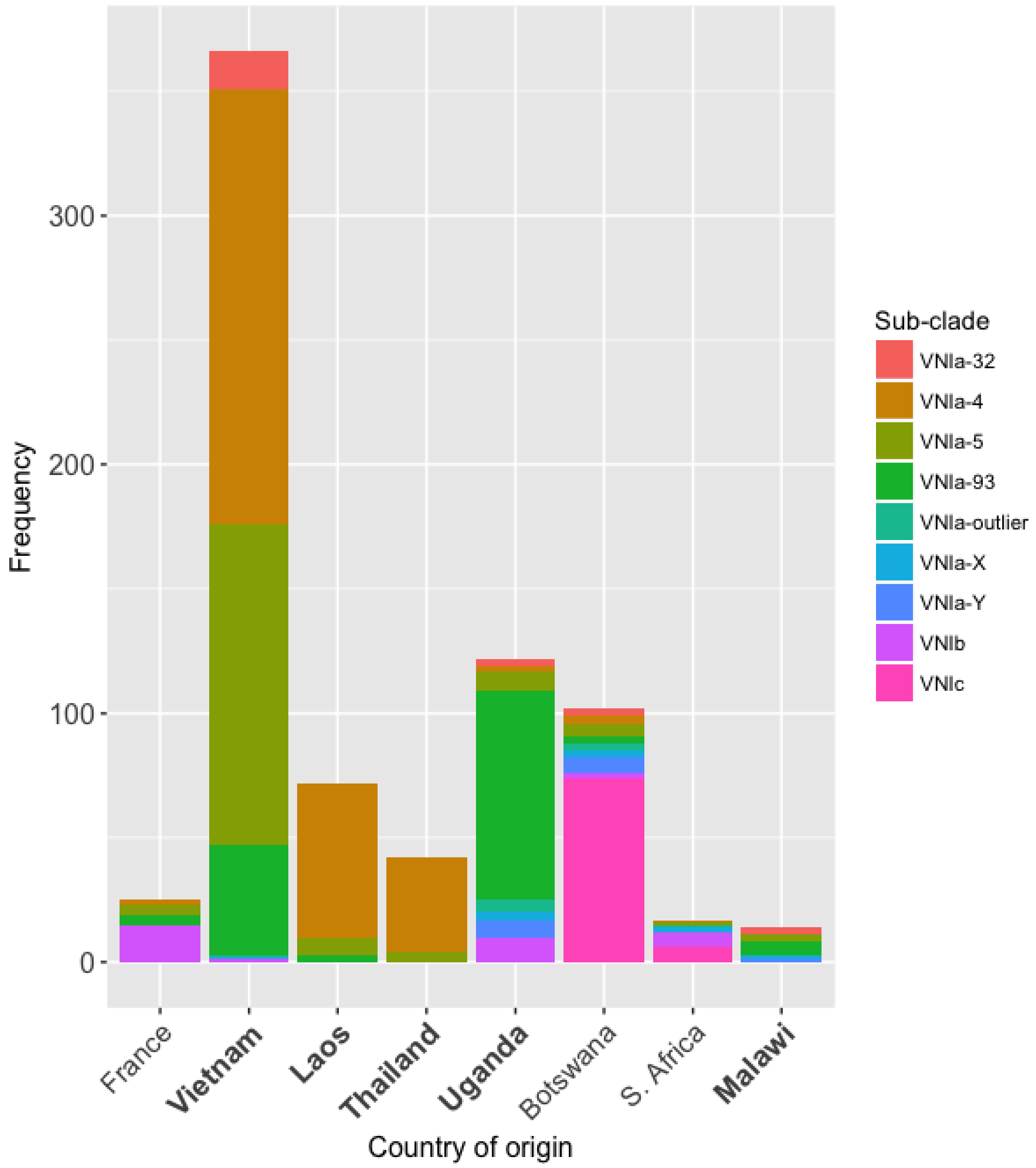

Supplementary Figure 4: Geographical distribution of the main sub-clades of *C. neoformans* VNI identified in 612 isolates from this study and 148 from Desjardins et al. Only isolates from HIV infected patients were included, and only countries with more than 5 isolates from HIV infected people are depicted (full table in supplementary). Countries where >=95% of isolates were sequenced in this study are Vietnam, Laos, Thailand, Uganda and Malawi.

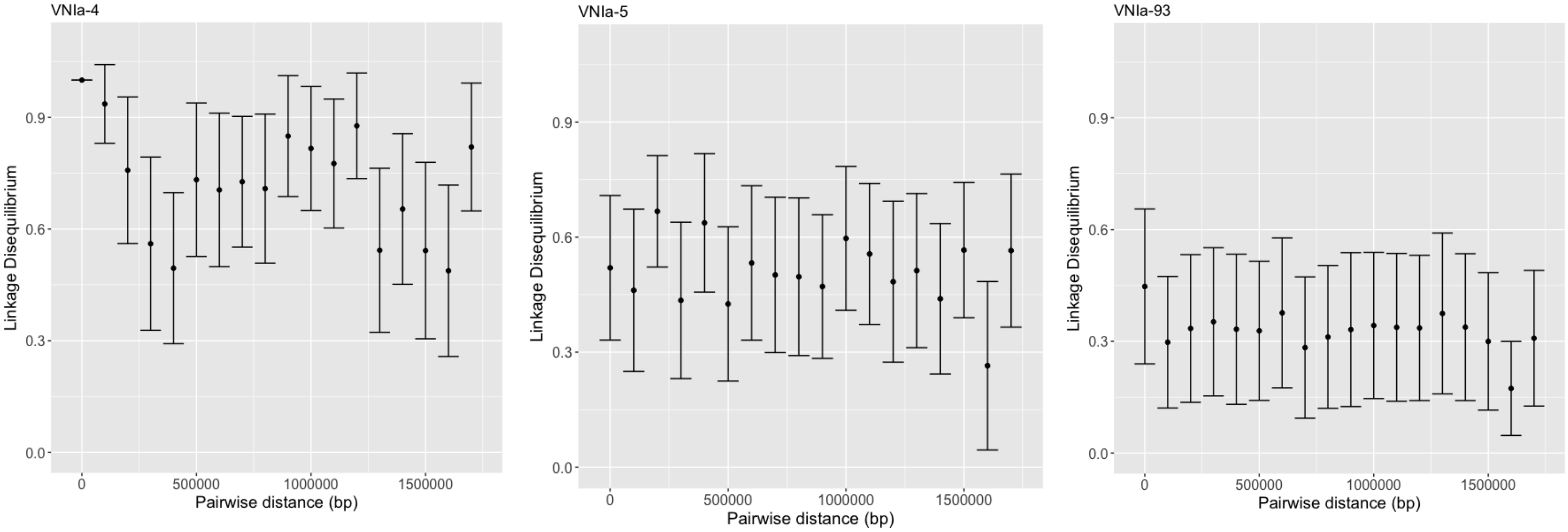

VNIa-4
VNIa-5
VNIa-93
Linkage Disequilibrium
Pairwise distance (bp)
0.0
0.3
0.6
0.9
0
500000
1000000
1500000

Supplementary Figure 5: Linkage disequilibrium in each sub-clade. LD was assessed using vcftools to generate R^2 for each pair of SNPs on the same chromosome. LD values were averaged over 100 kbp. Points on the graph represent mean LD while whiskers are standard deviation.

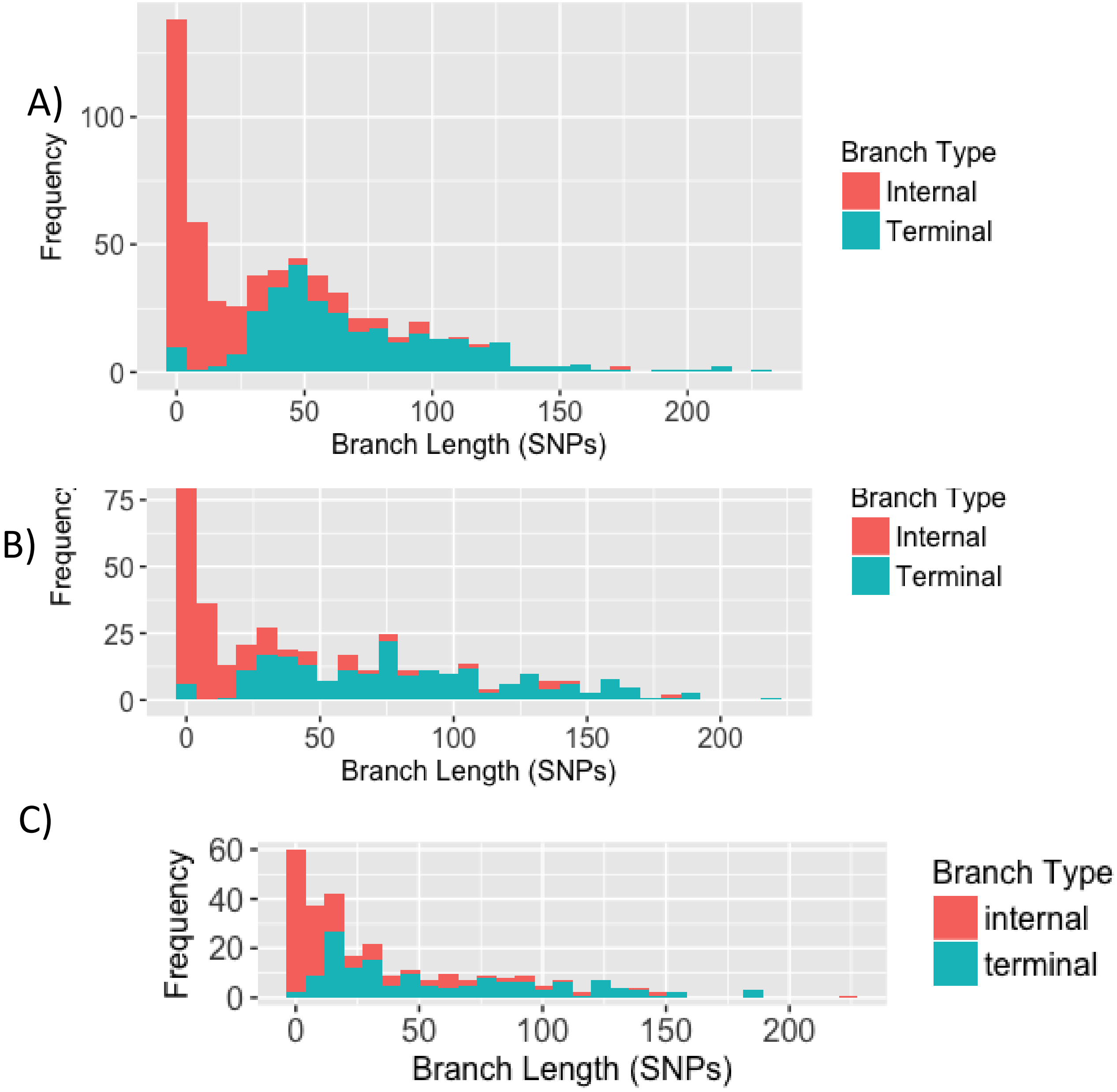

Supplementary Figure 6: The distribution of branch lengths in (A) VNIa-4 (B) VNIa-5 (C) VNIa-93. Differences between the internal and terminal branch lengths were statistically significant for all three sub-clades (P-value from Kolmogorov-Smirnov test less than $4x10^{-19}$).

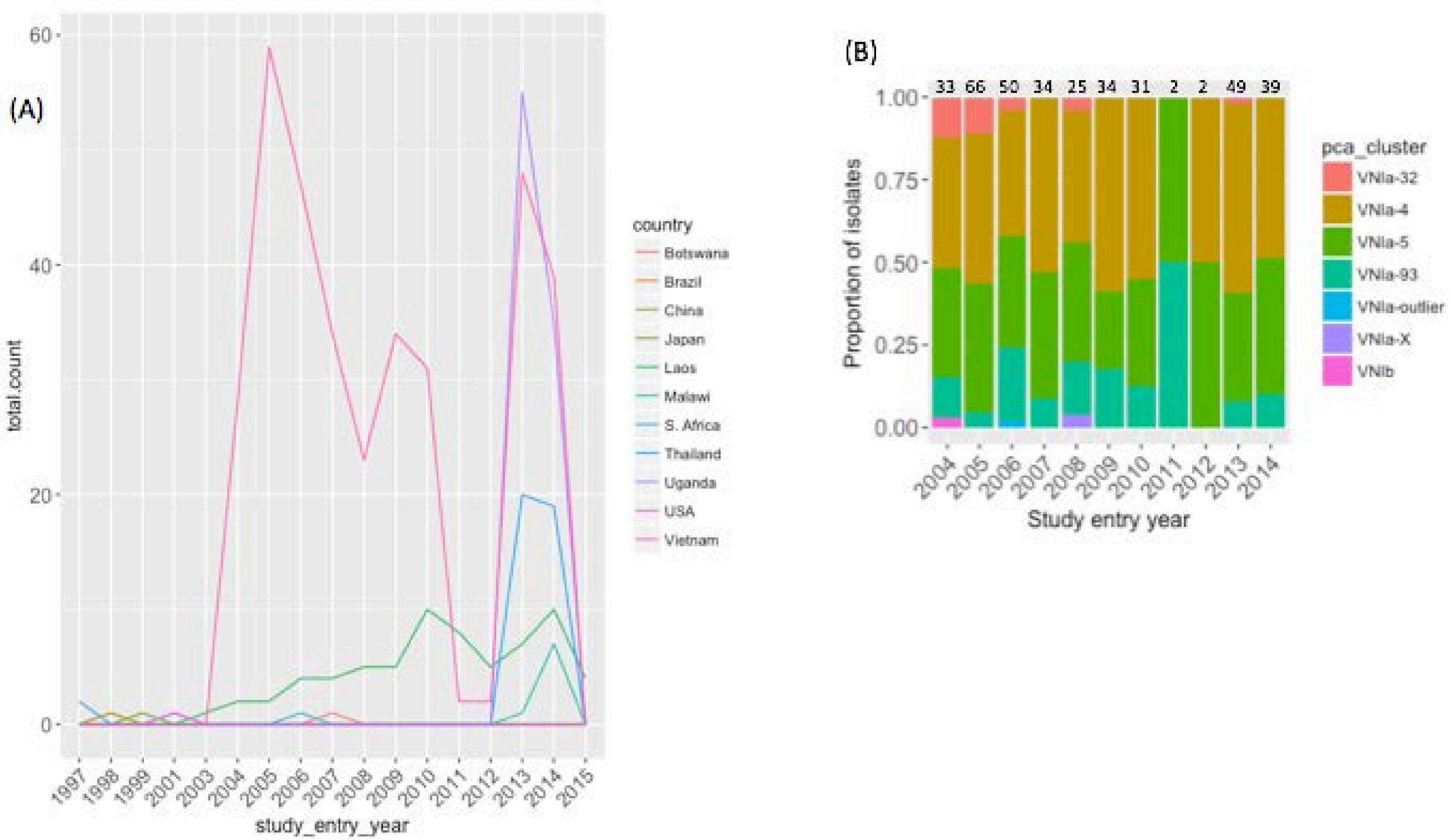

Supplementary Figure 7: The temporal distribution of C. neoformans collected as part of this study. (A) The total number of isolates collected between 1997-2015 in each country (B) The proportion of isolates of each lineage collected between 2004-2014 in Vietnam. The number of isolates collected per year is shown above each bar.

A)

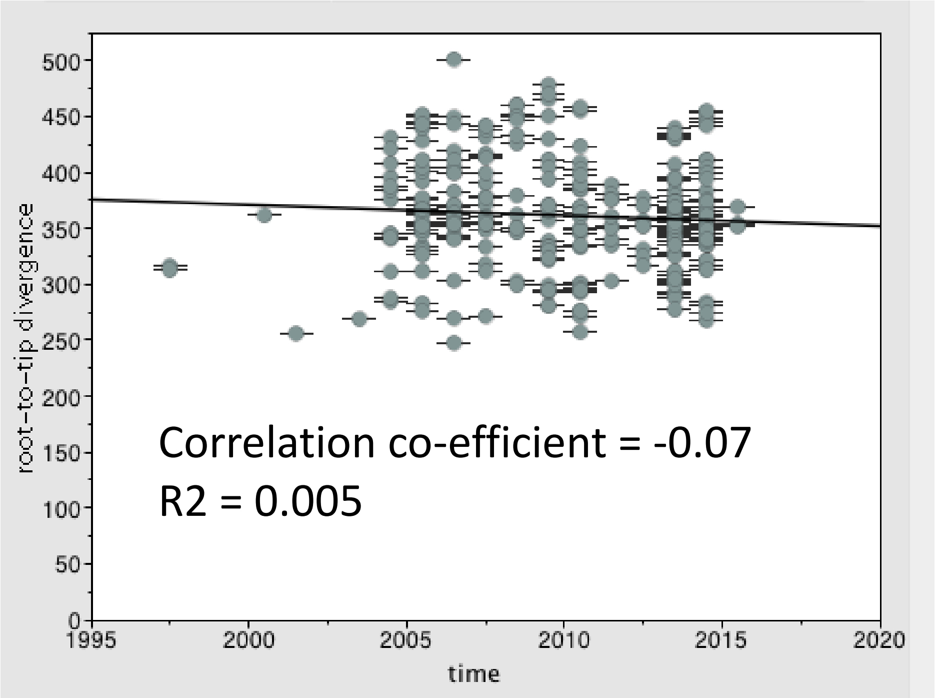
root-to-tip divergence
500
450
400
350
300
250
200
150
100
50
0
1995
2000
2005
2010
2015
2020
time
Correlation co-efficient = -0.07
R2 = 0.005

B)

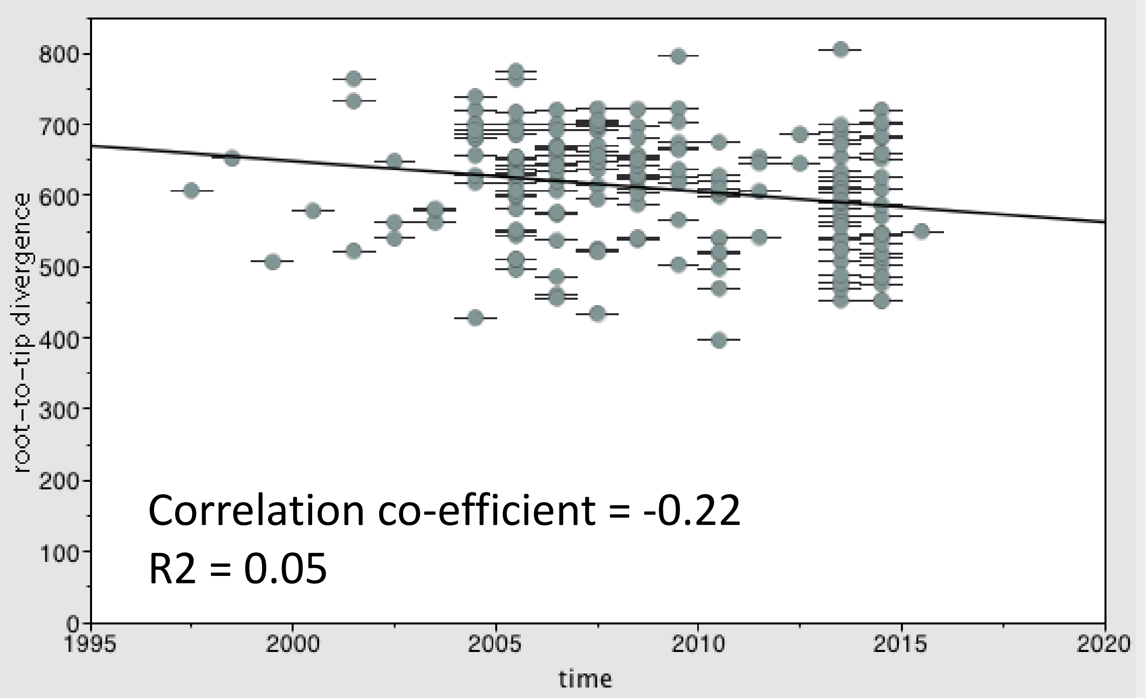
root-to-tip divergence
800
700
600
500
400
300
200
100
0
1995
2000
2005
2010
2015
2020
time
Correlation co-efficient = -0.22
R2 = 0.05

C)

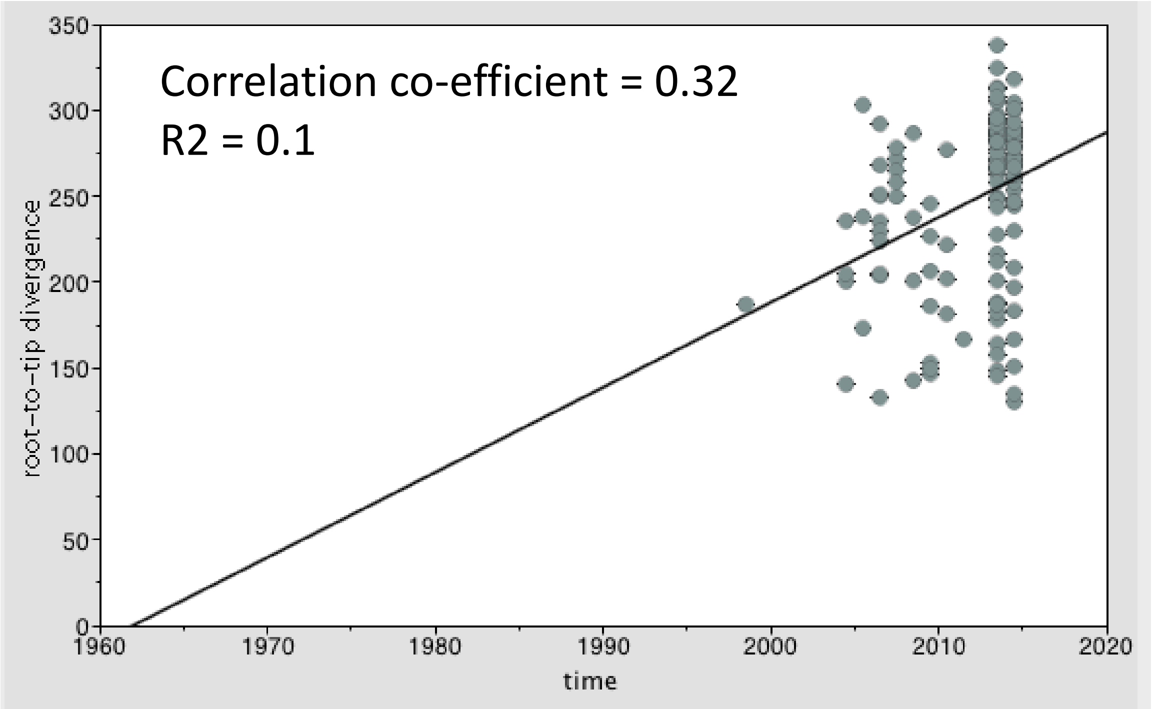

Supplementary Figure 8: The relationship between time of isolation and root to tip distance in A) VNIa-4 B) VNIa-5 C) VNIa-93

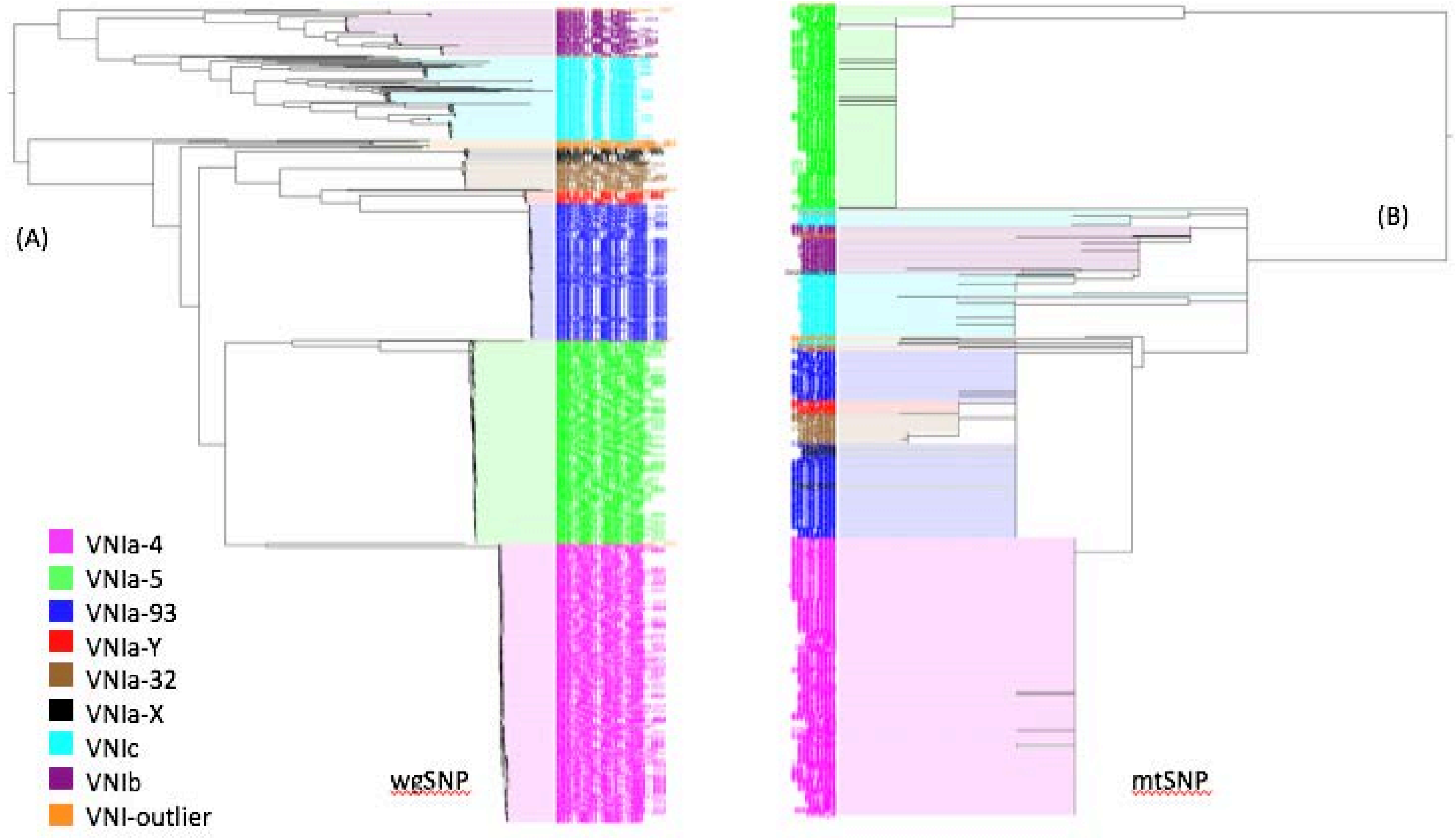

Supplementary Figure 9: Maximum likelihood phylogenies derived from variants in the whole genome (A) and in the mitochondria (B).

Supplementary Table 1: Distribution of clades by country, data from this study and Desjardins et al., 2017

Country	VNIa	VNIc	VNIb	Grand Total
Vietnam	430		1	431
Uganda	113		10	123
Botswana	29	83	3	115
Laos	73			73
Thailand	42			42
France	10		15	25
S. Africa	7	6	7	20
Malawi	13			13
USA	5		6	11
Togo			2	2
Tanzania	1			1
China	1			1
India	1			1
Australia			1	1
Brazil	1			1
Italy			1	1
Argentina			1	1
Japan	1			1
**Grand Total**	**727**	**89**	**47**	**863**

Supplementary Table 2: Distribution of clades by continent, data from both this study and Desjardins et al., 2017

Continent	VNIa	VNIb	VNIc	Grand Total
Asia	548	1		549
Africa	163	22	89	274
Europe	10	16		26
N. America	5	6		11
S. America	1	1		2
Australasia		1		1
**Grand Total**	**727**	**47**	**89**	**863**

Supplementary Table 3: Per sub-clade summary stats about polytomies

	VNIa-4	VNIa-5	VNIa-93
Number of polytomies	46	36	21
Number of collapsed branches (percentage of total branches)	78 (13%)	65 (15%)	35 (12%)
Median size	3	3	3
Max size	9	11	6
Max time range	10	15	8
Median time range	5.5	5	1
Number international	14	10	4
Number intercontinental	1	6	1

Supplementary Table 4: Results of Cox regression analysis for association between infection lineage and outcome at 10 weeks

	Variable	Hazard Ratio	Lower 95% Confidence interval	Upper 95% Confidence interval	p-value
**Infection Lineage***					
	VNIa-5	0.951	0.669	1.352	0.779
	VNIa-93	0.447	0.263	0.761	0.003
	Other	0.606	0.332	1.107	0.103
**Country$**					
	Thailand	1.456	0.872	2.432	0.151
	Laos	0.419	0.058	3.026	0.388
	Malawi	1.516	0.584	3.934	0.393
	Uganda	2.678	1.593	4.501	0
**Dexamethasone use#**					
	Dexamethasone use days 1-21	0.727	0.473	1.116	0.145
	Dexamethasone use dasy 22-70	2.801	1.516	5.173	0.001
**Induction Therapy@**					
	Amphotericin plus flucytosine	0.768	0.47	1.257	0.294
	Amphotericin plus fluconazole	0.847	0.551	1.304	0.451

* Compared with Lineage VNIa-4

$ compared with Vietnam

# Compared with no dexamethasone use

@ compared with amphotericin monotherapy

Supplementary Table 5: Results of Cox regression analysis for association between infection lineage and outcome at 6 months

	Variable	Hazard Ratio	Lower 95% Confidence Interval	Upper 95% Confidence Interval	P
**Infection Lineage***					
	VNIa-5	0.977	0.721	1.324	[illegible]
	VNIa-93	0.604	0.389	0.937	[illegible]
	Other	0.989	0.611	1.601	[illegible]
**Country$**					
	Thailand	1.476	0.913	2.386	[illegible]
	Laos	0.777	0.19	3.167	[illegible]
	Malawi	1.729	0.8	3.738	[illegible]
	Uganda	2.233	1.422	3.504	[illegible]
**Dexamethasone use#**					
	Dexamethasone effect days 1 to 21	0.762	0.499	1.164	[illegible]
	dexamethasone effect days 22 to 42	2.241	1.032	4.866	[illegible]

	dexamethasone effect days 43 to 180	2.741	1.512	4.968	[illegible]
**Induction Therapy@**					
	Amphotericin plus flucytosine	0.685	0.456	1.03	[illegible]
	Amphotericin plus fluconazole	0.709	0.497	1.01	[illegible]

* Compared with Lineage VNIa-4
$ compared with Vietnam
# Compared with no dexamethasone use
@ compared with amphotericin monotherapy

Supplementary Table 6: Premature stop codons identified in genes encoding DNA mismatch repair proteins

Position within chromosome	Reference base	Amino acid change	Variant base	Chromosome	Product	Locus	In which strain?
2024421	C	E201*	A	NC_026745.1	DNA mismatch repair protein MSH2	CNAG_00770	04CN-11-015
1270591	G	S240*	T	NC_026750.1	DNA mismatch repair protein MLH1	CNAG_02073	20427_3#29
1271452	G	Q10*	A	NC_026750.1	DNA mismatch repair protein MLH1	CNAG_02073	04CN-30-001
1271392	G	R30*	A	NC_026750.1	DNA mismatch repair protein MLH1	CNAG_02073	SRR1925902
439289	A	Y393*	C	NC_026748.1	DNA repair protein Rad5	CNAG_05102	SRR837099
217104	G	W697*	A	NC_026752.1	DNA cross-link repair 1A protein	CNAG_03160	04CN-03-102
