# Supplementary material for: Three phylogenetic groups have driven the recent population expansion of Cryptococcus neoformans
Source: Nat Commun. 2019 May 2;10:2035. doi: 10.1038/s41467-019-10092-5 (PMC6497710; doi:10.1038/s41467-019-10092-5)
Supplement: Supplementary file 4 — Description of Additional Supplementary Files [file 41467_2019_10092_MOESM4_ESM.pdf]

Description of Additional Supplementary Files

Supplementary Data 1: Line list of all Cryptococcus samples analysed in this study
